# The efficacy of *Cassia fistula* on constipation in chronic kidney disease patients in comparison with lactulose: A randomized clinical trial

**DOI:** 10.22038/ajp.2025.26012

**Published:** 2025

**Authors:** Iman Jahanian, Roghayeh Akbari, Hoda Shirafkan, Maedeh Rezghi, Seyyed Ali Mozaffarpur

**Affiliations:** 1 *Student Research Committee, Babol University of Medical Sciences, Babol, Iran*; 2 *Department of Internal Medicine, Babol University of Medical Sciences, Babol, Iran*; 3 *Social Determinants of Health Research Center, Health Research Institute, Babol University of Medical Sciences, Babol, Iran *; 4 *Traditional Medicine and History of Medical Sciences Research Center, Health, Research Institute, Babol University of Medical Sciences, Babol, Iran *; 5 *Department of Persian Medicine, School of Persian Medicine, Babol University of Medical Sciences, Babol, Iran*

**Keywords:** Cassia fistula, Chronic constipation, Chronic kidney disease, Persian Medicine, Lactulose

## Abstract

**Objective:**

Chronic constipation (CC) is common in chronic kidney disease (CKD) patients. This study was performed to evaluate the efficacy of *Cassia fistula* syrup (CFS) on CC in CKD patients.

**Materials and Methods:**

This randomized clinical trial was conducted on CKD patients with CC referred to nephrology clinics of Babol University of Medical Sciences in 2022-23.They were examined by a nephrologist and 66 patients were randomly allocated into two groups, receiving CFS or lactulose, with the same dosage (30 ml/day) for 2 weeks. Patients were visited in the 1^st^, 2^nd^ and 3^rd^ weeks to evaluate clinical characteristics according to ROME IV criteria, quality of life (PAC-QOL), and laboratory factor levels. Data were analyzed by SPSS25 using intention to treat viewpoint and by applying the ANCOVA, Repeated measure analysis, T-test and Chi-square test.

**Results:**

In the CFS group, defecation frequency per week and daily (primary outcome) improved significantly compared to the lactulose group (p-Value=0.01).

As secondary outcomes, the percent of straining, lumpy stools and stool hardness in the CFS group was significantly better than the lactulose (p <0.05). In the CFS group, blood urea nitrogen (BUN) (p-Value=0.045) and creatinine (Cr) (p =0.01) decreased after the intervention significantly. PAC-QOL showed significant improvements in subscales and total scores in the CFS group compared to the lactulose group (p<0.001).

**Conclusion:**

This is the first trial that evaluated CFS on CC in CKD patients, and monitored the changes in laboratory factors levels. CFS demonstrates greater efficacy than lactulose in managing CC among CKD patients. PAC-QOL was greatly better in CFS group rather than lactulose group.

## Introduction

Chronic kidney disease (CKD) is characterized by the existence of kidney damage or a continual decrease in glomerular filtration rate (GFR), defined as GFR<60 ml/min per 1.73 m^2^, persisting for a duration of three months or more, regardless of the underlying etiology (Bargman and Skorecki 2015). It is a state of progressive loss of kidney function, ultimately resulting in the requirem

ent for renal replacement therapy such as dialysis or transplantation (Vaidya and Aeddula 2024).

In CKD patients, especially in advanced stages, the prevalence of constipation is higher than in the general population (Strid et al. 2002).

The prevalence of this symptom ranges from 1.6 to 70.7% for hemodialysis patients and from 14.2 to 90.3% for peritoneal dialysis patients (Zuvela et al. 2018).

Low dietary fiber intake, inactivity, water restriction, concurrent drug use, decreased gastrointestinal motility and alterations in gut microbiota are common causes of constipation in CKD. Constipation has significant negative implications for overall health. Its presence has been notably related to the deterioration of kidney function, an elevated risk of progressing to advanced stages of CKD (Ruszkowski et al. 2020), and increased mortality in CKD patients (Sumida et al. 2020). Additionally, constipation is correlated with an increased prevalence of bone fractures in pre-dialysis patients with CKD and lower quality of life (QOL) scores in patients with end-stage renal disease (ESRD) (Yamada et al. 2023). Consequently, constipation leads to increased medical and socioeconomic burdens among CKD patients. Hence, effectively managing constipation is imperative to provide comprehensive care for CKD patients. 

Lifestyle modifications, dietary adjustments, and non-pharmacological and pharmacological interventions can be used to treat constipation (Cha et al. 2023).

Non-absorbable carbohydrates, including hyperosmolar laxatives like sorbitol, lactitol, and lactulose, are utilized in the treatment of constipation   (Tayebi-Khosroshahi et al. 2016) . Studies have indicated that lactulose induces alterations in gut microbiota, leading to the suppression of uremic toxin production and improvement in kidney function. Thus, lactulose has the potential for demonstrating renoprotective effects and tolerance in CKD patients. However, side effects including abdominal pain and distension, diarrhea, and abnormal gastrointestinal sounds can restrict its administration (Sueyoshi et al. 2019).

Complementary and alternative medicines are used in many countries to enhance health services and are often applied alongside conventional medicine to prevent, treat, and restore health (Organization 2019). Persian Medicine (PM), based on an individualized approach(Mojahedi et al. 2018) and the humoral theory, is one of the alternative and traditional medicines that focuses on lifestyle adjustments and the use of natural products such as medicinal plants, for patient management (MOZAFFARPUR et al. 2016; Mozaffarpur et al. 2020).

*Cassia fistula* is a prominent medicinal plant utilized in numerous traditional medicinal systems (Mwangi et al. 2021) and is broadly administered as a mild laxative in various patient groups within PM (Mozaffarpur et al. 2012a).

Based on studies, *C. fistula* has been shown to possess antioxidant, anti-inflammatory, antibacterial, wound healing, hepatoprotective, anti-diabetic, laxative (Mwangi et al. 2021), and anticancer properties (Remya et al. 2015) and additionally, it has been proven as a safe and cost-effective treatment against multidrug-resistant infections (Chakraborty et al. 2022).

In research, various species of *Cassia* such as *C. angustifolia*, *C. fistula *and* C. alata* have been found to be as efficient as conventional laxatives because of their anthraquinone derivatives (Aslam 2021).

In two clinical studies, *C. fistula* emulsion was greatly more effective than mineral oil (Mozaffarpur et al. 2012b) and as efficient as Poly Ethylene Glycol (PEG) in treating 3 weeks of pediatric functional constipation (Esmaeilidooki et al. 2016). In a clinical trial, the impact of *C. fistula* syrup (CFS) on constipation in 70 geriatric patients was examined. After two weeks, the frequency of defecation, the percent of straining, pain during defecation, the consistency of stool and QOL in CFS group were significantly better than the lactulose group (Sepehr et al. 2022).

Another clinical trial study was performed on 70 pregnant women with constipation. Participants were randomly divided into two groups. Both groups received dietary and physical activity recommendations. Additionally, *C. fistula* (CF) was prescribed for the intervention group. After a two-week intervention, the CF group exhibited a significantly higher frequency of defecation per week (Esmaeilzadeh et al. 2023). Also, in an animal study, the oral lethal dose 50% (LD50) of the aqueous extract of *C. fistula* was determined to be greater than 5000 mg/kg of body weight in rat. There were no notable changes in maternal blood urea or creatinine levels. The findings showed that the *C. fistula* could be considered safe during pregnancy (Hakiminia et al. 2022).

Given the limited research on constipation management in patients with renal failure and the critical need for a safe and effective treatment, this study was conducted to assess the efficacy of CFS on chronic constipation among patients with CKD, as compared to lactulose.

## Materials and Methods

### Trial Design

This randomized, parallel-arm (1:1) clinical trial was conducted in the nephrology clinics of Babol University of Medical Sciences (BUMS), Iran in 2022-23. The project was approved by research ethics committee (IR.MUBABOL.HRI.REC.1401.075) and then registered on IRCT (IRCT20200105046009N6). Written informed consent was filled out by all patients.

### Inclusion criteria

Patients with CKD, and CC based on ROME IV criteria, and aged between 18-80 years, were included in this study.

### Exclusion criteria

History of sensitivity to CFS, active infections in last month, surgery on the GI system, pregnancy, or organic constipation was the exclusion criteria.

### Dropout criteria

Complications or sensitivity to the medicine, misuse of the medication (those who have used less than 75% of the total amount of the medicine), and concurrent use of another laxative drug were considered dropout criteria.

### Entering to the study

CKD patients referred to the nephrology clinics at BUMS with CC were checked for eligibility criteria. After confirmation by a nephrologist, the patients entered to this survey and their demographic data, defecation frequency per week and ROME IV criteria within previous three months were documented.

### Intervention

Patients who met the eligibility criteria were randomly assigned to two study groups:

### Case group

In this study, CFS which contains the standard aqueous extract of *Cassia fistula *and is a product of Sanabel Daru Co. (Flobel) (batch No. 065-96-02), was applied. The preparation method was on the basis of the PM textbooks (Mozaffarpur et al. 2012b). The *Cassia fistula *pod pulp was isolated and boiled in water. The acquired extract was then combined with sugar and almond oil, and a gentle heating process was used to prepare the syrup.

CFS was standardized based on the amount of the anthraquinone content, aloin, which was 0.011 mg/ml based on the HPLC method; total phenolic content measured by Folin-Ciocalteu reagent using gallic acid was 4.1 mg/ml, total flavonoid content measured by aluminum chloride reagent using rutin was 1 mg/ml and density and pH of the CFS were 2.79 g/ml and 5, respectively. Total Aerobic Microbial Count was less than 10 CFU/ml and total yeast and mold count was less than 10 CFU/ml.

The dose of CFS was determined based on PM textbooks and previous clinical studies (Esmaeilzadeh et al. 2023; Sepehr et al. 2022) at 30 ml/day (One tablespoon containing 10 ml of CFS three times a day).

### Control group

Lactulose syrup, a product of Exir Co. (batch No.2123137258100001) was administered 30 ml/day, the same as CFS. 

### Primary outcome

Defecation frequency (per week and daily) was primary outcome.

### Secondary outcomes

Secondary outcomes were straining, incomplete emptying, manual maneuvers, the laboratory changes (blood urea nitrogen (BUN), creatinine (Cr), glomerular filtration rate (GFR), Na and K) and the QOL.

### Measurement tools

- Data gathering questionnaire on the basis of ROME IV criteria for frequency of each variable per day/week was applied.

In this study, the measurement tools for secondary outcomes were based on our previous clinical studies (Esmaeilzadeh et al. 2023; Sepehr et al. 2022).

- Laboratory changes: laboratory tests were performed according to nephrologist visits and recorded in a checklist at the beginning of the study and post-intervention (Bargman and Skorecki 2015).

- QOL: Patient Assessment of Constipation Quality of Life Questionnaire (PAC-QOL) at the beginning of the study and post-intervention (end of 2nd week).

The PAC-QOL questionnaire is the most valid and the most specific tool for measuring the quality of life (QOL) of patients with constipation (Marquis et al. 2005). The Persian version of the PAC-QOL questionnaire demonstrated good validity and reliability in chronic constipation and includes 28 items estimating the constipation impacts on QOL within recent 2 weeks. It is divided into 4 items on physical discomfort, 8 items on psychosocial discomfort, 5 items on treatment satisfaction, and 11 items on worries and discomfort (Nikjooy et al. 2018).

### Intervention and follow up

We treated patients for 2 weeks and followed up for 1 week. 

Patients were told to record the symptoms such as frequencies of defecations per day and per week, straining with defecations, lumpy or hard stools, feeling of anorectal obstruction/blockage, incomplete evacuation and manual maneuvers, within a daily form by themselves or with the help of a family member.

Additionally, they were inquired to define their pain and hardness of each defecation according to visual analog scale (VAS) in which the scores ranged between 0 (minimum) and 10 (maximum). 

PAC-QOL was filled out for all patients before and after the medication (end of 2nd week). 

### Sample size

Using G^*^Power 3.1.9.2 software for 0.05 of the 1st type error, 90% of the power, 0.25 of the effect size and the frequency of 4 measurements of the primary outcome for repeated measure analysis (evaluated at baseline, and in the 1st, 2nd and 3rd weeks), 30 patients in each group were calculated. Considering probability of dropout rate at 10%, we set the ultimate sample size at 33 patients per group. 

### Randomization and concealment process

We applied permuted block randomization method in blocks of size 4 including 2 of each A and B, ratio 1:1. Random sequence was produced by statistics and sample size application. Every package got a unique 4-digit code, and was delivered to the researcher blindly. After ending the study, unlocking the codes was done. Drug codes could be opened in the event of side effects.

In order to conceal the allocation, random digit codes were produced corresponding to each drug for each patient.

The codes were written on the CFS/lactulose packages. With entering each patient to the study, one package of CFS/lactulose was allocated due to randomization consequence, and the codes on the packages were written in the patient’s checklist.

### Blinding

The shape and color of packages of both CFS and lactulose were the same. The research pharmacist put CFS and lactulose syrup in the same packages and delivered to the statistician. The statistician, researcher, and outcome assessors were blind to the intervention. (Outcome assessor blind)

### Statistical methods

Data analysis was accomplished by SPSS25 and the significance level was considered less than 0.05.

We present descriptive statistics as mean±SD and frequency (%). Kolmogorov–Smirnov test was applied to assess the normality of data. To compare the parameters at baseline, we utilized chi-square and independent t-test. 

We applied intention to treat (ITT) analysis, and all the patients entered to the analysis. Multiple imputation method (EM algorithm) was used to impute the missing values. The trend of outcome changes was tested by general linear model (repeated measure analysis). Analysis of covariance (ANCOVA) was administered to assess the effect of the treatment on QOL. 

We used ANCOVA for QOL that was a quantitative variable. For other outcomes if they were quantitative, we used repeated measure analysis.

In ANCOVA analysis the dependent variable was the QOL assessment after the intervention, we included the intervention group variable as factor and the baseline assessment of QOL as covariate.

The secondary outcomes such as 'Manual maneuvers required', refer to the frequency of each mentioned variable, so they are quantitative variables and we used repeated measure analysis.

For comparing time duration to improvement between the two groups, we used Kaplan-Meier analysis and Log Rank test. The improvement of each patient was assessed by ROME IV criteria.

## Results

**Figure 1:**
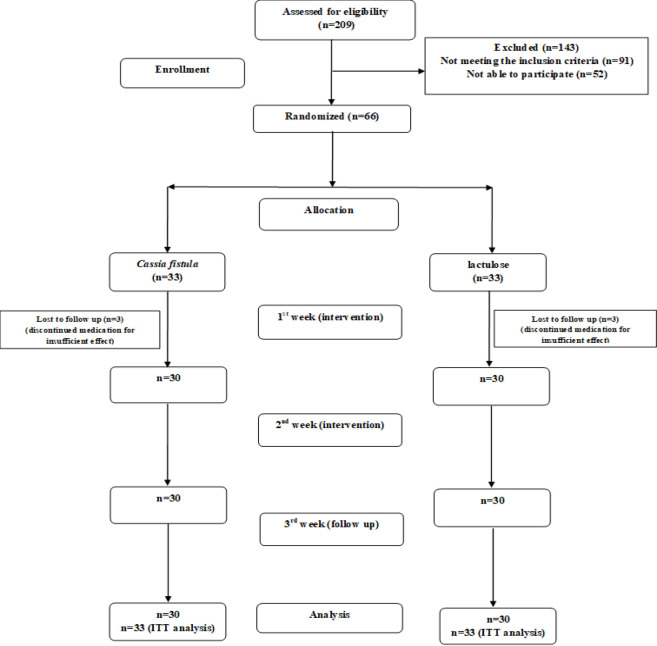
CONSORT flowchart

Out of the total 209 CKD patients, 66 patients with complaints of constipation met the eligibility criteria and were randomly divided into groups. The CONSORT flowchart for this survey is presented in Figure 1. The patient cohort exhibited a mean age of 68.63±7.59 years, with a range from 52 to 80 years. Among the participants, 34 individuals (51.5%) were female. In the baseline data analysis, no statistically significant differences were observed between the groups (p >0.05). Comprehensive details are mentioned in Table 1.

**Table 1:** Baseline characteristics of patients in both groups

**Variables**	**Mean±SD**		**p-Value** ^*a^
	***Cassia fistula*** **(n=33)**	**Lactulose** **(n=33)**	
Age (years)	67.66±8.19	69.60±6.94	0.30
Gender (female %)	51.5	51.5	1.00 ^*b^
Blood Pressure (Systolic)	128.78±20.88	126.66±26.18	0.71
Blood Pressure (Diastolic)	70.75±11.18	67.87±12.68	0.33
Pulse Rate (n)	74.06±10.31	75.66±12.39	0.56
Hemoglobin	12.11±1.88	11.56±2.27	0.29
Hematocrit	37.01±5.39	35.31±7.40	0.31
Platelets	213.45±44.75	246.65±145.08	0.22
Ca	9.5±0.56	9.35±0.55	0.42
P	4.45±0.83	4.03±0.77	0.13
GFR	33.36±11.98	34.54±13.42	0.70
Na	138.47±3.56	137.14±3.99	0.16
K	4.43±0.45	4.69±0.67	0.07
BUN	38.41±18.67	39.55±22.10	0.82
Cr	2.03±0.97	1.95±0.87	0.72

*a:Independent T-Test. *b: Chi-Square Test. *c: Ca (Calcium), P (Phosphorus), GFR (glomerular filtration rate), Na (Natrium), K (Kalium), BUN (blood urea nitrogen), Cr (creatinine).

### Primary outcome

In the CFS group, the defecation frequency per week ranged from 1.81±0.84 at baseline to 4.12±1.61 in the 1st week, 4.79±1.81 in the 2nd week, and 3.67±1.77 in the 3rd week of the study. This frequency was significantly higher than that observed in the Lactulose group, which varied from 2.09±0.87 at baseline to 3.69±1.69, 3.45±1.39, and 2.32±1.06, respectively (p =0.01). In this study, ROME IV criteria were used, so the frequency of primary outcome and percentage of secondary outcomes were considered. Additional information is presented in Table 2.

### Secondary outcomes

**Figure 2:**
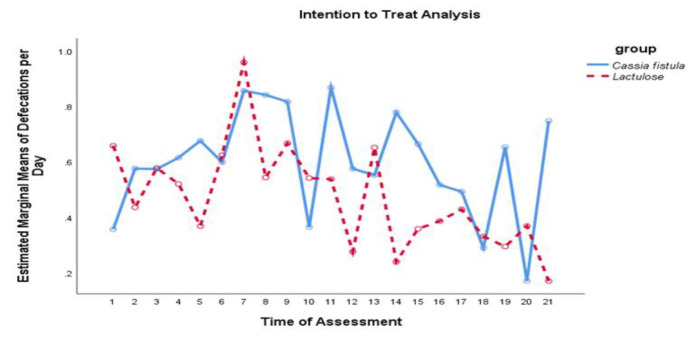
Comparing the effect of the intervention on the number of defecations per day during the treatment and follow up

Concerning the secondary outcomes of this study, both groups exhibited improvement in manual maneuvers required, sensation of anorectal obstruction/blockage, incomplete evacuation, and pain with defecation; however, no significant difference between the groups was observed (p>0.05). Notably, the CFS group demonstrated significantly better results than the Lactulose group in terms of defecation frequency per day (p =0.01) (Figure 2), defecation days per week (p =0.01), lumpy or hard stools (p =0.001), hardness of stools (p<0.001), and straining with defecations (p<0.001) (Table 2).

**Table 2:** Treatment outcomes and intervention effects on patients’ defecation status at the study's onset and post-intervention

**Variables**	**Group**	**Mean±SD**				p-Value*	**Partial Eta Squared**
		**Baseline**	**1st Week**	**2nd Week**	**3rd Week**		
Defecations per week (n)	CFS	1.81±0.84	4.12±1.61	4.79±1.81	3.67±1.77	0.01	0.08
	Lactulose	2.09±0.87	3.69±1.69	3.45±1.39	2.32±1.06		
Defecation days per week (n)	CFS	-	3.49±1.15	4.56±1.87	3.49±1.59	0.01	0.09
	Lactulose	-	3.45±0.89	3.32±1.45	2.32±1.06		
Straining with defecations (%)	CFS	86.36±31.30	43.85±31.84	23.31±31.94	51.22±49.67	< 0.001	0.20
	Lactulose	90.15±23.33	65.95±21.52	63.63±30.36	88.34±31.72		
Manual maneuvers required (%)	CFS	12.12±33.14	5.56±14.92	7.82±26.03	9.96±27.17	0.98	< 0.001
	Lactulose	10.60±29.33	6.59±19.64	9.53±26.11	9.19±27.88		
Lumpy or hard stools (%)	CFS	93.93±16.57	53.84±68.94	32.10±67.35	44.79±34.12	0.001	0.14
	Lactulose	97.45±8.27	77.79±43.31	64.20±33.94	89.83±20.14		
Sensation of incomplete evacuation (%)	CFS	18.18±39.16	7.32±25.04	4.45±33.45	5.01±23.12	0.73	0.002
	Lactulose	18.18±36.58	7.34±22.61	6.50±23.47	11.33±32.18		
Sensation of anorectal Obstruction/Blockage (%)	CFS	3.03±17.40	0.63±4.05	1.02±16.59	2.39±10.66	0.21	0.02
	Lactulose	11.36±31.30	2.73±7.72	3.26±11.18	6.32±16.53		
Hardness of Stools (VAS)	CFS	9.06±1.02	4.44±1.33	2.36±1.52	4.18±1.76	< 0.001	0.26
	Lactulose	8.18±1.01	5.99±1.37	4.78±1.65	6.70±1.52		
Pain with defecation (VAS)	CFS	3.48±0.90	1.40±0.73	0.39±0.62	1.94±0.84	0.09	0.04
	Lactulose	2.78±1.24	1.99±1.05	1.35±1.10	2.49±1.08		

* General Linear Model (repeated measure analysis)

Comparison of changes in laboratory factors level in the two groups at baseline and after the intervention reveals a significant decrease in BUN (p =0.045) and Cr (p =0.01) in the CFS group compared to the Lactulose group. However, GFR, Na, and K showed no significant differences (p>0.05) between the groups (Table 3).

As shown in Table 3, QOL exhibited a significant improvement in all four subscales and total PAC-QOL scores in the CFS group compared to the Lactulose group (p<0.001).

According to Figure 1, three patients in each group discontinued medication for insufficient effect in the 1st week of intervention. Information related to the CFS/ Lactulose side effects (at any time and with any quality) were recorded in a data gathering form. Also, about 1-2 phone calls were made a week to inform about the patient's condition. Patients were asked for different symptoms such as abdominal pain, nausea, vomiting, heartburn, indigestion, flatulence, diarrhea, allergic/skin reactions. No significant side effect was reported in the CFS/ Lactulose groups.

Our findings indicate that the time to treat was the first week in the CFS group and the third week in the Lactulose group.

According to ROME IV criteria, two or more elements of the criteria must be present for constipation diagnosis. If so, the patient is remained untreated after the medication, otherwise, the patient is considered treated.

**Figure 3:**
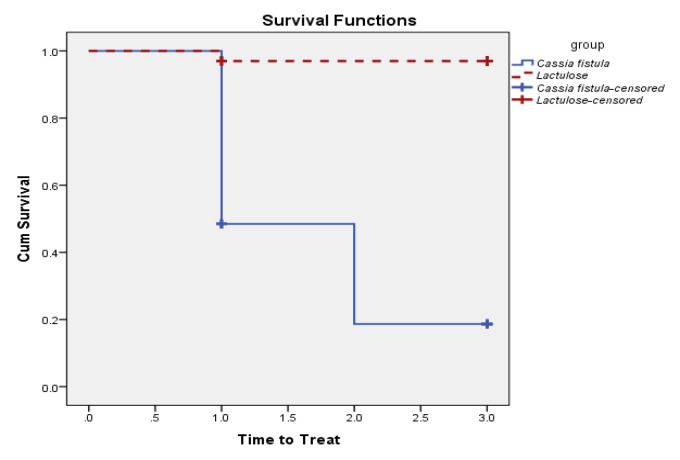
Comparison of survival functions and time to treat in CFS and Lactulose groups

According to the Kaplan-Meier model (Figure 3), the time to treat in the CFS group was significantly shorter than the Lactulose group (Log Rank p<0.001).

**Table 3:** The changes in laboratory factors levels and PAC-QOL at the beginning of the study and post-intervention

**Variables**	**Time**	**Mean±SD**		p-Value*	**Partial Eta Squared**
		**CFS**	**Lactulose**		
GFR	Baseline	33.36±11.98	34.54±13.42	0.059	0.055
	Post-Intervention	35.80±11.98	33.61±14.20		
Na	Baseline	138.15±3.66	136.92±3.96	0.22	0.023
	Post-Intervention	139.22±3.04	137.78±3.75		
K	Baseline	4.43±0.45	4.69±0.67	0.99	< 0.001
	Post-Intervention	4.50±0.44	4.62±0.48		
BUN	Baseline	38.41±18.67	39.55±22.10	0.045	0.062
	Post-Intervention	34.65±16.63	40.71±23.29		
Cr	Baseline	2.03±0.97	1.95±0.87	0.01	0.09
	Post-Intervention	1.87±0.77	2.02±0.84		
Physical Discomfort	Baseline	11.39±2.16	9.87±2.36	< 0.001	0.44
	Post-Intervention	3.97±2.89	7.05±2.72		
Psychosocial Discomfort	Baseline	13.97±3.01	11.75±3.01	< 0.001	0.44
	Post-Intervention	6.09±1.93	8.49±2.99		
Worries and Discomfort	Baseline	25.60±2.24	23.15±3.76	< 0.001	0.54
	Post-Intervention	9.57±4.62	16.83±3.86		
Satisfaction	Baseline	17.69±2.54	16.54±2.01	< 0.001	0.29
	Post-Intervention	6.35±4.69	11.03±3.43		
Total	Baseline	68.66±6.07	61.33±7.04	< 0.001	0.47
	Post-Intervention	25.99±12.26	43.43±10.11		

* ANCOVA

### Per-protocol method analysis

In per-protocol method, the findings are as follows that are similar to ITT method:

### Primary outcome

In the CFS group, the defecation frequency per week ranged from 1.83±0.87 at baseline to 4.13±1.65 in the 1st week, 5.10±1.39 in the 2nd week, and 3.90±1.34 in the 3rd week of the study. This frequency was significantly higher than that observed in the Lactulose group, which varied from 2.16±0.87 at baseline to 3.80±1.73, 3.70±1.05, and 2.50±0.86, respectively (p =0.01).

### Secondary outcomes

Concerning the secondary outcomes of this study, both groups exhibited improvement in manual maneuvers required, incomplete evacuation, and pain with defecation; however, no significant difference between the groups was observed (p>0.05). Notably, the CFS group demonstrated significantly better results than the Lactulose group in terms of defecation frequency per day (p =0.007), defecation days per week (p<0.001), lumpy or hard stools (p<0.001), hardness of stools (p<0.001), sensation of anorectal obstruction/blockage (p=0.04), and straining with defecations (p<0.001).

Comparison of changes in laboratory factors levels in the two groups at baseline and after the intervention reveals a significant decrease in BUN (p=0.03) and Cr (p=0.050) in the CFS group compared to the Lactulose group. However, GFR, Na, and K showed no significant differences (p>0.05) between the groups.

QOL exhibited a significant improvement in all four subscales and total PAC-QOL scores in the CFS group compared to the Lactulose group (p<0.001).

## Discussion

In this randomized, parallel-group clinical trial study, the efficacy of CFS for treating CC in patients with CKD was compared to lactulose in terms of defecation frequency, laboratory factor levels, and QOL.

In this study, improvement in CFS group rather than Lactulose group was identified as large effects on frequency of straining with defecations, lumpy or hard stools, hardness of stools and PAC-QOL scores (Partial Eta Squared>0.11). Meanwhile, intermediate effects were observed on defecation frequency per week, defecation frequency per day, number of defecation days, BUN and Cr (0.039<Partial Eta Squared<0.11). Partial Eta Squared is a measure of effect size indicating the proportion of variance in the dependent variable that is associated with the independent variable.

CC is one of the most prevalent gastrointestinal disorders referred to medical professionals (Jamshed et al. 2011). It is often supposed that CC in CKD patients has been managed by physicians based on clinical expertise and/or the use of general therapeutic agents (Xing and Soffer 2001), or has remained untreated as a common, ignorable problem. Some CKD patients consider constipation self-manageable and never seek medicinal follow-up. Constipation is a frequent symptom in patients with CKD, negatively impacting their QOL and potentially contributing to excess morbidity and mortality. This is attributed to the association between constipation and gut dysbiosis which can lead to the accumulation of gut-derived uremic toxins as renal function declines. The presence of constipation in CKD may limit the gut's ancillary roles, further exacerbating the condition (Ikee et al. 2020; Mugie et al. 2011).

*Cassia fistula *L. has been widely used in traditional medicine as a safe, mild laxative for various groups of patients including children and pregnant women. In previous studies, CFS appeared to be efficient in improving defecation frequency, pain and stool consistency in pediatric constipation compared with mineral oil (Mozaffarpur et al. 2012b) and PEG (Esmaeilidooki et al. 2016). A clinical trial study in pregnant women proved that CFS was more efficient in controlling constipation compared with dietary plus physical suggestions (Esmaeilzadeh et al. 2023).

According to Ade et al., Ayurveda has clearly mentioned that *C. fistula *is one of the drugs frequently used for the treatment of constipation. Ade et al. selected 31 patients with constipation who were treated by *C. fistula* granules, twice a day. Follow-up was done and modified ROME-III questionnaire was utilized to assess the effect of therapy and was filled out before and after completion of treatment. Statistically significant results observed in most studied parameters (Ade et al. 2018).

In another clinical trial, 36 Thai patients with constipation according to Rome III Criteria were randomized into 2 groups. Main outcome was stool frequency, consistency and ease of evacuation. No significant difference was observed in laxative functions between *C. angustifolia and C. fistula*. Ethnologically, *C. fistula *has long been applied as a laxative because of its safety and low cost, so could be developed as Senna, a commercial laxative (Mangmeesri et al. 2014).

In a quasi-experimental survey on 26 female aged 18-25 years old with constipation, *C. fistula *showed significant effectiveness on a decrease in severity of the constipation measured by constipation scoring system (Jannah et al. 2017).

Based on our best knowledge, this study is the first survey that evaluated CFS in CKD patients. 

Also, we performed the laboratory tests including BUN, Cr, Na and K as part of our nephrologic care to ensure patient safety and monitor potential side-effects. Comparison of changes in laboratory factor levels in the two groups at baseline and after the intervention reveals a significant decrease in BUN and Cr in the CFS group compared to the Lactulose group. 

Monitoring kidney function is crucial for maintaining overall health, and BUN and Cr are valuable screening tests. They serve as useful markers for tracking the progression of the renal disease. The results revealed that CFS can significantly reduce serum Cr and BUN levels in CKD patients, suggesting potential renal protective effects. Many studies have shown that anthraquinone compounds found in plants can prevent and treat CKD and exhibit pharmacological activity in alleviating renal dysfunction (Li et al. 2017; Zhang et al. 2023). However, the specific mechanism of action of CFS in reducing laboratory factors such as BUN and Cr has not been investigated in studies, and further investigations are needed to explore this potential relationship.

Nowadays, health care systems try to manage not only the symptoms but also the QOL after treatment, which is a prominent point to assess therapeutic agents (Marquis et al. 2005), so we have precisely conducted the same approach in our survey.

In a recent study on geriatric constipation, CFS proved to be more effective than lactulose. The defecation frequency per week, percent of straining, hard stool, pain, stool consistency and QOL were significantly better in CFS group. The feeling of incomplete evacuation, anorectal obstruction, and manual maneuvering were not different statistically (Sepehr et al. 2022). Our study confirmed that the total score of PAC-QOL (and its subscales) indicated significant improvements in CFS group compared with Lactulose group.

The study of Shirvani Farsani et al. in patients undergoing hemodialysis, showed that there is a significant relationship between the PAC-QOL and its subscales with the severity of constipation (Shirvani et al. 2023).

In a multicenter and observational study by Kamei et al., the Japanese PAC-QOL was used on 27 patients who met the Rome 3 criteria for CC (at baseline and after 4 weeks). Hemodialysis patients are prone to CC, which can adversely affect their QOL and Elobixibat (a highly selective inhibitor of the ileal bile acid transporter, can subsequently enhance colonic motility and secretion) may have a novel mechanism for treatment that had not been reported. The number of spontaneous bowel movements increased significantly. Significant decreases in the PAC-QOL scores were noticed (Kamei et al. 2020).

A clinical trial was performed by Lydia et al. on 60 patients with CKD on hemodialysis (CKD-HD) with GI complaints, difficult defecating, and hard consistency. Gut microbiota dysbiosis causes an increase in proteolytic bacteria activity, leading to producing more uremic toxins, worsening of constipation and reducing QOL. This study found a great effect in improving constipation symptoms and QOL after symbiotic administration (Lydia et al. 2022).

Totally, our findings are supported by the results of the above researches.

In the present study, CKD patients experienced significant relief from constipation when treated with *C. fistula*, a plant rich in anthraquinones, which has been found to have laxative properties (Bahorun et al. 2005; Hakiminia et al. 2022; Iyengar et al. 1966). Anthraquinone compounds are broken down into active molecules by intestinal flora which increase the water and electrolytes and improve intestinal motility by stimulating the myenteric neural network (Dan et al. 2013). The purgative effect of the anthraquinone in *C. fistula *happens through two separate methods. First mechanism includes changes in colon motility, resulting in a quick large intestinal transit. Changes in motility occur indirectly due to high demand within epithelial cells. Second mechanism includes modification of the colonic absorption associated with secretion leading to fluid accumulation and then diarrhea (Gritsanapan 2010).

In our study, the treatment period was two weeks, which is consistent with previous investigations on CFS (Esmaeilidooki et al. 2016; Mozaffarpur et al. 2012b). This herbal syrup has been well tolerated during this period. Despite the potential hypokalemic impacts of anthraquinones-containing herbs (Emeriau et al. 1983), we did not observe any specific side-effects during follow-up assessments. However, further research is clearly needed to comprehensively assess the safety profile of CFS.

One of the strengths of our study is that we stayed in touch with patients regularly by phone calls once or twice a week. We informed the patients about the study process, completing the forms and possible complications that might happen. In addition, from the practical viewpoints, lactulose has less side-effects than other common laxatives and is simply accessible at a fair expense (Ford and Suares 2011), so we have decided to utilize it for the control group as a strong competitor. Furthermore, we used the PAC-QOL as a worldwide used assessment questionnaire (Mokhtare et al. 2017).

We compared all demographic (age, gender), clinical (blood pressure and pulse rate), laboratory characteristics (BUN, Cr, GFR, Na, K …), and the baseline of primary and secondary outcomes at the baseline of the study. We found no significant difference between groups in baseline characteristics which implies CFS efficacy is determined without any confounding factors. As the randomization process was completely run and the randomization method was permuted blocks, we controlled the effect of confounders. This investigation may support a growing trend to traditional medicine treatments including PM in recent years.

The short intervention time was a limitation of the present survey. In CC, the recurrence of the symptoms after the intervention is probable. Additionally, for long-term administrations, side-effects of CFS must be considered. 

As constipation is common in CKD patients and no drug of choice exists, more efforts in new researches are still essential to improve CC management in CKD patients, therefore, a long-term medication and follow-up, more laboratory testing, and life style changes are also suggested for future studies. Conducting applied academic interventions seems necessary to develop evidence-based information resources for physicians. We believe that our study findings will be of interest to the researchers of this field.

In conclusion, this is the first trial that evaluated CFS on CC in CKD patients, and monitored the changes in laboratory factors levels such as BUN, Cr, Na and K. Overall, our findings showed that CFS is more effective than lactulose on CC in CKD. Among the laboratory tests, BUN and Cr showed significant decrease in CFS group. Also, PAC-QOL was greatly better in CFS group rather than Lactulose group.
